# Trends in procedures for infertility and caesarean sections: was NICE disinvestment guidance implemented? NICE recommendation reminders

**DOI:** 10.1186/1471-2458-13-112

**Published:** 2013-02-06

**Authors:** Charlotte A Chamberlain, Richard M Martin, John Busby, Rebecca Gilbert, David J Cahill, William Hollingworth

**Affiliations:** 1 School of Social and Community Medicine, University of Bristol, Canynge Hall, 39 Whatley Road, Bristol BS8 2PS, UK; 2 Centre for Reproductive Medicine, Division of Obstetrics and Gynaecology, University of Bristol, St. Michael’s Hospital and Centre for Medical Education, First Floor South, Senate House, Tyndall Avenue, Bristol BS8 1TH, UK

**Keywords:** NICE, Clinical guidelines, Fertility, Caesarean

## Abstract

**Background:**

National Institute for Health and Clinical Excellence (NICE) clinical guidelines and subsequent NICE issued ‘recommendation reminders’ advocate discontinuing two fertility procedures and caesarean sections in women with hepatitis. We assess whether NICE guidance in 2004 and recommendation reminders were associated with a change in the rate of clinical procedures performed.

**Methods:**

Routine inpatient Hospital Episode Statistics (HES) data were extracted from the HES database for 1st April 1998 to 31st March 2010 using OPCS procedure codes for varicocele operations in infertile men, endometrial biopsies in infertile women and caesarean sections in women with hepatitis B or C. We used Joinpoint regression to identify points in time when the trend in procedure rates changed markedly, to identify any influence of the release of NICE guidance.

**Results:**

Between 1998-2010, planned caesarean sections in women with and without hepatitis B or C increased yearly (annual percentage change (APC) 4.9%, 95% CI 2.1% to 7.7%) in women with hepatitis, compared with women without (APC 4.0% [95% CI 2.7% to 5.3%] up to 2001, APC -0.6% [95% CI -2.8% to 1.8%] up to 2004 and 1.3% [95% CI 0.8% to 1.8%] up to 2010). In infertile women under 40 years of age, endometrial biopsies for investigation of infertility increased, APC 6.0% (95% CI 3.6% to 8.4%) up to 2003, APC 1.5% (95% CI -4.3% to 7.7%) to 2007 followed by APC 12.8% (95% CI 1.0% to 26.0%) to 2010. Varicocele procedures remained relatively static between 1998 and 2010 (APC -0.5%, 95% CI -2.3% to 1.3%).

**Conclusions:**

There was no decline in use of the three studied procedures, contrary to NICE guidance, and no change in uptake associated with the timing of NICE guidance or recommendation reminders. ‘Do not do’ recommendation reminders may be ineffective at improving clinical practice or achieving disinvestment.

## Background

National Institute for Health and Clinical Excellence (NICE) recommendation reminders identify practices that should be discontinued where they are not evidence based. These reminders, also known as optimal practice reviews and the basis of the newer ‘do not do’ guidance database, are drawn from NICE cancer service guidance, clinical guidelines, interventional procedures and technology appraisals guidance, and aim to help the NHS make ‘better use of its resources [1].’ As of 2012, there were 147 NICE recommendation reminders (issued from 2000 to 2006) extracted from 34 different NICE clinical guidelines and technology appraisals. Fertility is the clinical area with the highest number of recommendation reminders issued (22). Five reminders pertain to caesarean section clinical guidelines. After the 2004 fertility and caesarean section NICE clinical guidelines (clinical guidelines 11 and 13, respectively), the following recommendation reminders were released: (i) caesarean sections are not indicated to reduce the transmission of hepatitis B or C from mother to child [2]; (ii) the use of endometrial biopsies in the luteal phase to investigate female infertility is not indicated [3]; (iii) the use of varicocele operations as a means to treat infertility in men is not indicated [3]. The caesarean section guideline has been updated (Nov 2011) with no changes to the above recommendations.

The impact of these recommendation reminder prompts has never been formally evaluated, although it is clear that there is variable adherence to NICE clinical guidelines and technology appraisals in a range of clinical practice areas [4-10]. This paper assesses the impact of four NICE recommendation reminders on the use of three procedures, which should be discontinued according to the evidence base. Our hypothesis is that NICE clinical guidelines and reminders should precede a reduction in the trend of procedure uptake, in line with the best evidence.

## Methods

Trends in procedure volume were assessed using routine, freely available, English inpatient patient-level Hospital Episode Statistics (HES) data [11] from the NHS information Centre from 1st April 1998 to 31st March 2010 (each HES year commences on 1st April and ends 31st March) using relevant procedure codes (OPCS 4) stratified by the designated ICD-10 diagnosis codes (Table 1) in all diagnoses. We included both primary and secondary procedure and diagnosis codes and included all episodes during a hospital spell. We computed rates of planned and unplanned caesarean sections per 100,000 deliveries, using the total number of deliveries (OPCS-4 codes: R17 to R25) [12] and women with hepatitis B and C combined. We compared trends in caesarean section rates in women with and without hepatitis over time using women without hepatitis as an observational ‘control’. Similarly we compared trends in unplanned and planned caesarean section rates over time considering trends in the general population.

**Table 1:** ICD-10 and OPCS-4.5 procedure codes for HES data on recommendation reminders

**Recommendation reminder**	**Publication date of original guidelines**	**ICD-10 code & description**	**OPCS-4 procedure code & description**
**Caesarean Section:**	**April 2004**	**B16: Acute hepatitis B**	**R17: Planned caesarean**
**Recommendation reminder 3:** There is insufficient evidence that planned caesarean reduces mother-to-child transmission of hepatitis B virus when transmission can be reduced by administration of immunoglobulin and vaccination to the child.		**B180: Chronic viral hepatitis B with delta- agent**	**R18: ‘Other’ caesarean**
		**B181: Chronic viral hepatitis B without delta-agent**	
**Caesarean Section:**	**April 2004**	**B182: Chronic viral hepatitis C**	**R17: Planned caesarean**
**Recommendation reminder 4:** Women who are infected with hepatitis C should not be offered a planned caesarean because this does not reduce mother-to-child transmission of hepatitis C virus		**B171: Acute hepatitis C**	**R18: ‘Other’ caesarean**
**Fertility:**	**February 2004**	**N97: Female Infertility**	**Q18 Diagnostic endoscopic examination of uterus**
**Recommendation reminder 1:** Women should not be offered an endometrial biopsy to evaluate the luteal phase as part of the investigation of fertility problems			**Q10.8: curettage of uterus-other specified**
			**Q20.2:biopsy of lesion of uterus nec**
**Fertility:**	**February 2004**	**N46: Male infertility**	**N19: procedures on a varicocele- specifically**
**Recommendation reminder 9:** Men should not be offered surgery for varicoceles as a form of fertility treatment			**N19.1: Ligation of**
			**N19.2: Embolisation of**
			**N19.8: Other specified**
			**N19.9: Unspecified**

We calculated the annual rate of endometrial biopsies per 100,000 finished in-patient consultant episodes (FCEs) for infertile women. The rates of endometrial biopsy in women with a primary diagnosis of infertility were stratified by age above or below 40 years (> 40, ≤ 40), as the primary indication for endometrial biopsies above age 40 may have been investigations to rule out endometrial cancer, rather than for luteal phase infertility. Women with missing ages (1.8% of women under 40) were excluded from the analysis. We conducted a sensitivity analysis to explore the impact on our conclusions of excluding women without a primary diagnosis of infertility, where endometrial biopsies may have been undertaken for an alternative indication.

The rate of varicocele procedures per 100,000 finished consultant episodes in infertile men was calculated per annum. We included male infertility recorded in either primary or secondary diagnosis codes (up to 23 secondary diagnosis codes) as exploratory analysis revealed that some primary diagnosis codes were related to the procedure itself (e.g. varices).

Joinpoint regression software (version 3.5.2 http://surveillance.cancer.gov/joinpoint/) was used to identify points in time when the trend in procedure rate changed markedly, a so called ‘join point’. The annual rates of procedure use over time were calculated as an annual percentage change [APC]). The joinpoint analysis was based on Poisson rates and allowed calculation of 95% confidence intervals around the joinpoint date and the APC. Joinpoint software compares models with sequential hypothesis testing using permutation tests and Bayesian Information Criterion to generate different numbers of join points and to determine the best-fit data series [13]. The joinpoint data were assessed by testing the null hypothesis of no difference between the slope of each trend of annual rate of change segment against the previous neighbouring segment. To identify the line of best-fit with joinpoint the null and alternative hypotheses significance levels are varied to achieve an overall significance level of 0.05 (5%). We accounted for multiple testing using an amended Bonferroni correction [13].

## Results

### Caesarean section in women with hepatitis

**Figure 1:**
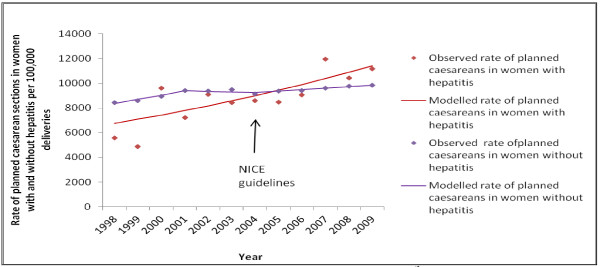
Planned caesarean section deliveries in women with and without hepatitis B or C, compared with guidance release.

There were 5,546 births to women with either hepatitis B or C between 1998 and 2010, of which 3,712 were normal vaginal deliveries, 829 unplanned caesarean sections, 525 planned caesarean sections and the remainder were instrumental, breech or unknown delivery method. There were 10 caesareans in women with hepatitis B or C in 1998, compared with 93 in 2010; the model-based estimates suggest that the underlying planned caesarean section rate in women with hepatitis B or C increased by 4.9% annually (APC 4.9%, 95% CI 2.1% to 7.7%). Unplanned caesarean sections in women with hepatitis B or C increased at a slightly lower average of 2.9% per annum (95% CI 0.3% to 5.6%) i.e. three more unplanned caesarean sections per 100 deliveries each year. There was no evidence of a change in the underlying trend of increased uptake of planned or unplanned caesarean section delivery in women with hepatitis when the NICE guidance was published in 2004, nor at any time between 1998 and 2010 (Figure 1). Results were similar when hepatitis B and C positive rates were calculated separately.

Women without hepatitis also experienced an overall increased number of planned caesarean section deliveries, from 43,619 in 1998 to 63,007 in 2010. Women without hepatitis had a lower annual model-based increase in planned caesarean sections overall, compared to women with hepatitis: APC 4.0% (95% CI 2.7% to 5.3%) up to 2001, APC -0.6% (95% CI -2.8% to 1.8%) up to 2004 and APC 1.3% (95% CI 0.8% to 1.8%) up to 2010. Rates for unplanned caesarean sections in women without hepatitis demonstrated a more gradual rise in procedures over the twelve year period compared both with unplanned caesarean sections in women with hepatitis and planned caesarean sections in women without hepatitis. The model-based average annual percent change was 1.9% (95% CI 1.6 to 2.2) up to 2007, followed by a decreasing rate up to 2010 (APC -1.3%, 95% CI -4.2 to 1.8). The estimated year where the change in trend occurred (joinpoint) could have occurred as early as 2004 or as late as 2007 (95% CI, p = 0.046).

### Endometrial biopsy for female infertility

**Figure 2:**
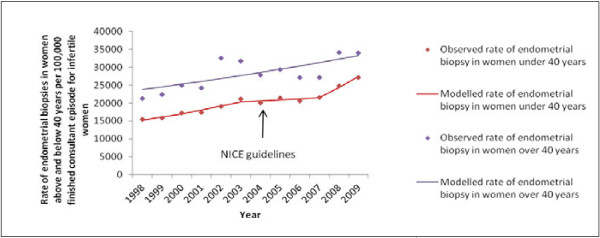
Endometrial biopsies in women > and < 40 years of age out of total finished consultant episodes for infertility, compared with the timing of NICE fertility guidelines.

Out of 137,028 admissions for female infertility (primary diagnosis), 26,527 endometrial biopsies were performed (19%) between 1998 and 2010. Although the number of endometrial biopsies was higher in women under 40 years, (mean per year 2,210) compared with those over 40 years (mean per year 338) the rate of procedures was lower (15,430 per 100,000 compared with 21,356 per 100,000 admissions, respectively in 1998). In women under 40 years, there is an absolute reduction in the number of endometrial biopsies per year (2,859 to 2,162) over the period; however, due to a concurrent reduction in the number of admissions in this group, there was an increase in the rate of endometrial biopsies over time. The increasing rate fluctuated from an APC of 6.0% (95% CI 3.6% to 8.4%), up to 2003 (with biopsy numbers declining from 2859 to 2141); a slower increase of APC 1.5% (95% CI -4.3% to 7.7%) from 2003 to 2007; and a faster rise in biopsies, APC 12.8% (95% CI 1.0% to 26.0%) between 2007 and 2010 (Figure 2). The 95% confidence interval around the estimated year at which change occurred suggests that the slowing of the upward rate in procedures in 2003 could have occurred between 2000 and 2004 (95% CI, p-value 0.025) and the rapid upward deflection in rate could have occurred as early as 2005 (although the upper confidence interval remained at 2007, p-value 0.05).

211,928 women with endometrial biopsies (79%) had infertility as their primary diagnosis code, with remaining primary diagnosis codes including recognised contributory causes of infertility (pelvic adhesions 5,772 (2.1%), endometriosis 3,103 (1.4%), polycystic ovarian syndrome 3,825 (1.4%) leiomyoma 3,570 (1.3%), in vitro fertilisation 3,805 (1.4%) and other diagnoses (all <1% each)). Sensitivity analysis in women, where infertility was coded as either a primary or secondary diagnosis, showed similar trends in procedure rates.

### Varicocele procedures for male infertility

**Figure 3:**
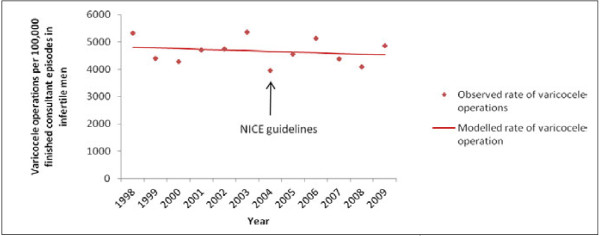
Varicocele operations in infertile men compared with the timing of NICE fertility guidelines.

Out of 9,399 finished consultant episodes for male infertility (all diagnoses), 437 (4.6%) underwent varicocele procedures over the twelve year period. There was no appreciable change in procedure volume between 1998 and 2010 (Figure 3), starting from 49 procedures in 1998 to 48 in 2010, with 5 fewer procedures for every 1000 men seen in hospital for infertility per annum (APC -0.5%, 95% CI -2.3% to 1.3%). Specifically, there was no change in trend after NICE guidance.

## Discussion

### Main findings

The observed changes in the procedure rates over time were not consistent with the decline in rates that would be expected if English NHS trusts had responded to the NICE “do not do” guidance. Between 1998 and 2010, the proportion of deliveries performed by planned caesarean section increased in all women, with a steeper increase in women with hepatitis, against best-practice guidance.

Numbers of male varicocele procedures were low and changed little during the study. The low procedure rate may be a reflection of many clinicians being aware of, and largely complying with, best practice before NICE guidance was issued. However, as a ‘do not do’ guidance, a decline even in the already low rate would have been anticipated. Endometrial biopsy rates, on the other hand, have shown an unexpected general increase in women less than 40 years, with only a transient slowing between 2003 and 2007. This increase is present in both women above and below 40 years and when infertility was used in primary and secondary diagnosis codes. This increase is contrary to guidance and shows no association with the timing of the fertility guidance. Overall, there is no good evidence that the release of the reminders had any effect on the trends in clinical practice for any of the procedures studied.

### Strengths and limitations

This work should be interpreted in light of some limitations, including difficulties extrapolating what trends would have occurred in procedure rates had guidance not been issued. We have partially overcome this for the caesarean section analysis by looking at women without hepatitis as an observational ‘control’ group. In addition, by assessing three very different procedures we hope to reduce the risk of attributing change to guidance that may be secondary to other secular trends. Any change in procedure trend, would not necessarily be caused by NICE guidance, only temporally associated with it. We are also unable to detect whether individual NHS trusts are enacting NICE guidance, relying instead on nationally collected evidence, which has shown that the guidance in these clinical areas has not appreciably influenced the trend in procedure performance. Any inference from this national data to the local trust level would be subject to ecological fallacy. Since the intention of the recommendation reminders and ‘do not do’ guidance is to effect a reduction in clinical procedure uptake, however, we can conclude, based on our study, that the guidelines appear not to have had an effect, since none of the procedures declined appreciably.

NICE does not record the date when recommendation reminders are published, which is an additional limitation, but since most observed changes in practice occurred before the NICE clinical guidance release (February and April 2004) and therefore, certainly before the recommendation reminder which follows the guidance, this would not alter our interpretation. There were two exceptions where there were changes in trend after guidance was issued: endometrial biopsies, which saw an increase in rate in 2007, the opposite of guidance and the fall in unplanned caesareans in women without hepatitis, which was not the focus of the caesarean guidelines or recommendation reminders. We are unable to account for any changes over time in the accuracy of diagnostic coding, but the consistency of our findings across three different procedures suggests that our results and interpretation are probably not due to coding anomalies changing over time.

More specific limitations, relevant to the clinical problems, include the fact that a caesarean section that occurs in a woman with hepatitis may not be solely because of the hepatitis B or C, but for a valid clinical reason, such as a breech presentation. We have therefore compared rates of planned and unplanned procedures in women with and without hepatitis to extrapolate, at an ecological level any trend to offset this concern. While the hepatitis C recommendation is a blanket ‘do not do’ guidance, the recommendation reminder for hepatitis B advises against planned caesarean section in women with hepatitis B on the presumption that the neonate will receive vaccination and immunoglobulin after birth. It is unlikely, therefore that a planned caesarean would be arranged to prevent mother-to-child transmission of hepatitis B in the UK, where the vaccine and immunoglobulin are freely available after birth.

To assess the quality of the HES data recording, we have compared the number of pregnant women with hepatitis B based on antenatal screening, with the number of hepatitis positive pregnant women in HES. The total number of deliveries in women with hepatitis coded in HES data was lower than the expected number of women with hepatitis B, based on antenatal screening (hepatitis B prevalence at antenatal clinic: 0.35% 2008; women delivering with hepatitis according to HES data: 0.11% in 2008) [14]. This raises questions as to the completeness of HES recording. This potential under-recording in HES could only affect our results if real reductions in caesarean section rates amongst women with hepatitis B were masked because recording of hepatitis B in women with caesarean section dramatically improved after 2004. The total numbers of women giving birth coded in HES is comparable with national epidemiological surveillance figures over the time period.

Our results show two procedures which have clear upward trends in procedure uptake, despite guidance to the contrary. The increasing trend in endometrial biopsies in women with infertility cannot be explained by competing evidence outside the published guidelines since there is good consensus in the peer-reviewed literature that endometrial biopsies are an ineffective means of predicting fertility and therefore, should not be conducted outside a research context [15]. We have attempted to control for artefactual explanations for the rise in endometrial biopsies by stratifying women into above and below 40 years and by limiting the analysis to only those women with infertility as their primary diagnosis, in case the biopsies are indicated for cancer investigation rather than infertility. Despite best evidence, the continued rise in planned caesarean section rates in women with hepatitis, compared to women without hepatitis, illustrates the potential difficulties in implementing disinvestment in this and other clinical guidelines where it is possible clinicians may dispense with NICE guidance, in favour of alternative evidence sources, such as published systematic reviews or local experience to dictate best practice. Unlike the consensus in endometrial biopsy indications amongst experts and the literature, there has been some debate in the literature for caesarean sections in women with hepatitis [16]. However, the updated NICE guidelines (2011) [17] uphold the original position that planned caesarean sections are not indicated for hepatitis status alone.

This work provides a clear evaluation of trends in three sub populations, in which procedures were identified as areas for potential disinvestment. Successful disinvestment from ineffective care is crucial if the NHS is to respond successfully to the current pressures on public funding. Our analysis uses twelve years of HES data [18] to describe changes in clinical practice that might be associated with NICE guidance for evidence based practice. Previous publications evaluating the implementation of NICE guidance, both those commissioned by NICE [8] and reported independently [10] were in 2005 and 2004 respectively and therefore, this is a timely update in light of the changing responsibilities of NICE [19]. The past literature focuses on NICE ‘technology appraisals’, which historically carried a mandatory funding requirement for commissioners, rather than the non-mandatory clinical guidelines [8,10]. Numerous evaluations of NICE implementation have revealed ‘under implementation’ of guidance [4,8]. Of those specifically assessing disinvestment decisions (two of the 45 implementation uptake reports) one has shown a decline in accordance with NICE guidance [20], the other a continued increase in drug prescribing, despite guidance [21]. Therefore, NICE may not only produce an insufficient number of disinvestment guidelines [22], but also have minimal evidence of their implementation.

## Conclusions

Our finding that the three studied procedures are not decreasing in line with NICE recommendations indicates a persistent research question around how best to implement NICE guidance and in particular, NICE disinvestment advice. Despite an increasing effort on behalf of NICE to introduce guidance implementation tracking, and the introduction of programmes to assist NHS Trusts in implementing evidence-based recommendations, there is still no clear steer on how to translate best evidence guidelines into best practice [23-25]. Clinical practice decisions are influenced by a number of factors including relevant literature, clinical guidelines, peer or senior support, as well as the policy environment [10,23-25]. The failure to implement recommendation reminders, which highlight areas for disinvestment, has particular implications both for clinical and cost effectiveness. Further study to supplement our understanding of other barriers to disinvestment could include the use of mixed methodology study with qualitative interviews exploring clinician engagement with disinvestment guidelines, and audit and prescribing data, which would counteract some of the missing data in the HES database [26]. Recommendation reminders have no demonstrable association with clinical practice nationally for the three procedures evaluated in this study and therefore, they may be an ineffective means to bring about disinvestment and change across England.

## Competing interests

The authors declare that they have no competing interests.

## Authors’ contributions

WH conceived of the study, participated in its design and coordination and revised the draft manuscript. DC participated in the study design, providing clinical advice and contributed to the drafting of the manuscript. JB updated all data extracts and participated in the analysis of the data and contributed to the draft manuscript. RG instructed CC in the use of joinpoint analysis, supervised its use, and contributed to the manuscript. RM participated in the design and coordination of the study, supervised the statistical analysis and helped to draft the manuscript. CC participated in the study design, carried out the analysis and drafted all versions of the manuscript. All authors read and approved the final manuscript.

## Pre-publication history

The pre-publication history for this paper can be accessed here:

http://www.biomedcentral.com/1471-2458/13/112/prepub
